# Checklists for Surgical Safety in Dermatosurgery

**DOI:** 10.4103/0974-2077.53090

**Published:** 2009

**Authors:** Venkataram Mysore, BS Anitha

**Affiliations:** *Venkat Charmalaya - Centre for Advanced Dermatology, Bangalore, Karnataka, India*

Surgical deaths and complications are a global public health problem. In the developed world, approximately half of all morbidity and mortality events affecting patients in hospitals are related to surgical care and services. It is possible that at least many of these morbidity and mortality events can be prevented if standards of care are adhered to and safety tools, such as checklists, are used.

Dermatology is no longer a pure medical specialty. It encompasses dermatologic surgery and dermatologists now perform advanced surgeries such as liposuctions, vitiligo surgeries, resurfacing, facelifts, cancer surgery etc. While most of these are done under local anesthesia, some of them may need general anesthesia. Further, a high degree of standard of care is needed for dermatologic surgical procedures as many of them are esthetic and are done in a previously healthy patient. Possibilities of medico-legal situations and esthetic complications always exist and therefore dermatologic surgeons should adopt checklists and standards of care in their practice. In this regard, it is important to note that the taskforce on dermatosurgery, of the Indian Association of dermatologists, venereologists and leprologists has published guidelines for standards of care in different dermatosurgical procedures.[1]

In June 2008, the World Health Organization (WHO) announced the “safe surgery saves lives” (SSSL) initiative to reduce surgical error and thereby promote patient safety.[1] The aim of the WHO Surgical Safety checklist is to ensure that key safety elements are incorporated into the operating room. Because of the simplicity and wide applicability, this checklist can be implemented in all countries irrespective of their economic status. While some of these guidelines may not apply fully to cutaneous surgical procedures, effective implementation of such similar systems by dermato-surgeons will go a long way in preventing complications during and after surgical procedures.

The SSSL initiative includes both the pre and peri-operative safety checklist and consists of three phases [Table 1].[2]

**Table 1:** WHO SSSL Pre and peri-operative safety checklist[2]

Sign in	Time out	Sign out
(Before induction of anesthesia)	(Before skin incision)	(Before the patient leaves operation theatre)
Patient has confirmed	Confirm all team members have introduced themselves by name and role	Nurse verbally confirms with the team
Identity		
Site		The name of the procedure recorded
Procedure		
Consent		That instrument, sponge and needle counts are correct (or not applicable) How the specimen is labeled (including the patient name) Whether there are any equipment problems to be addressed
Site marked/not applicable	Surgeon, anesthesia professional and nurse verbally confirm	Surgeon, anesthetist, and nurse review the key concerns for recovery and management of this patient
	Patient	
	Site	
	Procedure	
Anesthesia safety check completed	Anticipated critical events	
	Surgeon reviews: What are the critical or unexpected steps, operative duration, anticipated blood loss?	
	Anesthesia team reviews: Are there any patient-specific concerns?	
	Nursing team reviews: Has sterility been confirmed?	
	Are there equipment issues or any concerns?	
Pulse oximeter on patient and functioning	Has antibiotic prophylaxis been given within the last 60 min?	
	Yes	
	Not applicable	
	Is essential imaging displayed?	
	Yes	
	Not applicable	
Does patient have a known allergy		
Yes		
No		
Difficult airway/aspiration risk?		
Yes, and equipment/assistance available		
No		
Risk of >500 ml blood loss (7 ml/kg in children)		
No		
Yes, and adequate intravenous access and fluids planned		

This checklist has been formulated with the following objectives:[3]

1. To operate on the correct patient at the correct site.
2. To use methods known to prevent harm from anesthetic administration, while protecting the patient from pain.
3. To recognize and effectively prepare for life-threatening loss of airway or respiratory function and high blood loss.
4. To avoid inducing an allergic or adverse drug reaction known to be of significant risk to the patient.
5. To use methods known to minimize risk of surgical site infection.
6. To prevent inadvertent retention of sponges or instruments in surgical wounds.
7. To secure and accurately identify all surgical specimens.
8. To effectively communicate and exchange critical patient information for the safe conduct of the operation.
9. To establish routine surveillance of surgical capacity, volume and results.

These checklists have been shown to improve collaborative teamwork, minimize surprises, and lead to a safer day in the operating theater (OT).

In January 2009, the SSSL group published the results of a multicenter study, examining the impact of the implementation of the SSSL checklist system in eight centers (in India, Canada, USA, UK, Jordan, NZ, Tanzania and Philippines). The study demonstrated a significant decrease in postoperative deaths (1.5% versus 0.8%) and serious complications (11% versus 7%) as compared to those before the application of the checklist.[4]

These are the days of aesthetic practice; the eagerness to learn and practice aesthetics seems to be taking precedence over traditional dermatology and clinical skills. It seems more important these days to learn injecting a filler than to learn management of pemphigus. Being a cosmetologist seems more fashionable than being a dermatologist. “Doing” a treatment seems to be more important than “Why am I doing it?”. Hearsay seems to be more important than evidence. In addition to formulating and following surgical safety checklists, an ethical checklist should also be followed in aesthetic practice.

Such a checklist would include:

1. Always remember: Medicine is a science first, art next and commerce last of all.
2. Dermatological surgery is dermatosurgery, aesthetics and lasers. Dermatological surgery is evolving and evolving rapidly, very rapidly. So, reading, training and practice are very important.
3. Remember: Dermatosurgery needs staff, lasers need expensive instrument, aesthetics need expensive consumables-but all need skill. Do not invest in something you cannot afford, your EMIs should not drive your practice.
4. Aggressive treatment with training and experience is dynamism. Aggression without these is foolhardiness. Do not try to do what you cannot do! Do not do to others, what you won't do to yourself!
5. Do not try unproved treatment; view what is promoted by the companies with skepticism and seek proof. Do not experiment at a patient's expense.
6. Do not oversell yourself! Always under-promise and over-deliver.
7. *In medical practice, money should always be a byproduct - never the end product*. Practice ethical medicine. Integrity, honesty, and ethics are the cornerstones of success.
8. *Your most troublesome patient is your most important patient.* If any patient is unhappy with the treatment, see him/her for free and treat as a VIP patient. Do not avoid him! See him more often. Even if he is unhappy with your management, he should remember you as a good, sincere doctor who did his best.
9. Always prepare a separate consent form for each procedure.
10. *Those who forget history are condemned to repeat it.* Learn from mistakes. Never forget the roots, the basics. Do not ignore basic dermatology, dermatopathology. Vitiligo, psoriasis and eczemas are as important as a patient for hair removal or a filler. We are healers first and healers last!
